# Lupin Protein Concentrate as a Novel Functional Food Additive That Can Reduce Colitis-Induced Inflammation and Oxidative Stress

**DOI:** 10.3390/nu14102102

**Published:** 2022-05-18

**Authors:** Joana Mota, Sandra Casimiro, João Fernandes, Renata M. Hartmann, Elizângela Schemitt, Jaqueline Picada, Luís Costa, Norma Marroni, Anabela Raymundo, Ana Lima, Ricardo Boavida Ferreira

**Affiliations:** 1 LEAF—Linking Landscape, Environment, Agriculture and Food, Instituto Superior de Agronomia, Universidade de Lisboa, Tapada da Ajuda, 1349-017 Lisbon, Portugal; jcfernandes@isa.utl.pt (J.F.); anabraymundo@isa.ulisboa.pt (A.R.); agusmaolima@gmail.com (A.L.); rbferreira@isa.utl.pt (R.B.F.); 2 Faculty of Veterinary Medicine, Lusófona University, 1749-024 Lisbon, Portugal; 3 Clinical and Translational Oncology Research Unit, Instituto de Medicina Molecular João Lobo Antunes, Faculdade de Medicina de Lisboa, Universidade de Lisboa, 1649-028 Lisbon, Portugal; scasimiro@medicina.ulisboa.pt (S.C.); luiscosta.oncology@gmail.com (L.C.); 4 Laboratory of Experimental Hepatology and Gastroenterology, Hospital de Clínicas de Porto Alegre, Porto Alegre 90040-060, Brazil; renata.minuzzo@yahoo.com.br (R.M.H.); elizschemitt@yahoo.com.br (E.S.); nmarroni@terra.com.br (N.M.); 5 Genetic Toxicologic Laboratory, Lutheran University of Brazil (ULBRA), Canoas 92425-900, Brazil; jnpicada@gmail.com; 6 Oncology Division, Hospital de Santa Maria, Centro Hospitalar Universitário Lisboa Norte, 1649-028 Lisbon, Portugal

**Keywords:** colitis, HT29, IBD, lupin, antioxidant, MMP-9, cookies, functional food

## Abstract

Food fortification with bioactive compounds may constitute a way to ameliorate inflammatory bowel diseases (IBDs). Lupin seeds contain an oligomer named deflamin that can reduce IBD’s symptoms via MMP-9 inhibition. Here, our goal was to develop a lupin protein concentrate (LPC) enriched in deflamin and to test its application as a food additive to be used as a functional food against colitis. The nutritional profile of the LPC was evaluated, and its efficacy in vivo was tested, either alone or as added to wheat cookies. The LPC presented high protein and carbohydrate contents (20.09 g/100 g and 62.05/100 g, respectively), as well as antioxidant activity (FRAP: 351.19 mg AAE/10 mg and DPPH: 273.9 mg AAE/10 mg). It was also effective against TNBS-induced colitis in a dose dependent-manner, reducing DAI scores by more than 50% and concomitantly inhibiting MMP-9 activity. When added to cookies, the LPC activities were maintained after baking, and a 4-day diet with LPC cookies induced a significant protective effect against acetic acid-induced colitis, overall bringing lesions, oxidative stress and DNA damage levels to values significantly similar to controls (*p* < 0.001). The results show that the LPC is an efficient way to deliver deflamin in IBD-targeted diets.

## 1. Introduction

With the alarming growing incidence of chronic diseases worldwide, functional foods that exhibit health-promoting effects and can be safely used to reduce or prevent symptoms are receiving a lot of attention for certain types of pathologies such as gastrointestinal disorders, particularly inflammatory bowel diseases (IBDs), which include ulcerative colitis (UC) and Crohn’s disease (CD) [1]. These have long been recognized as a global burden, with prevalence rates of almost 250 cases per 100,000 persons, which are increasing worldwide in individuals of all ages [2]. Symptomatology includes mucosal inflammation, abdominal pain, diarrhea, bleeding and morphological changes in the colon such as epithelial erosion, edema formation and leukocyte infiltration (cryptitis) [3], which severely reduce the patients’ life quality [4]. The only available pharmacological treatments for IBDs are anti-inflammatory or immunosuppressive drugs, which are not only insufficient and show low-to-no effect for temporary symptoms, but are also prone to severe side effects [2]. To add to this, they are also expensive, which is a barrier for many patients, particularly for long-term therapies [2]. Therefore, it is unsurprising that the use of natural products to fight IBDs, particularly functional foods, is gaining worldwide attention. Indeed, diet itself is increasingly recognized as one of the most important influencing factors [5,6]. Whilst a wide range of dietary factors have been studied and recognized as driving the onset of inflammation, with certain food items being considered important triggers for IBDs [5,6], functional food efficacy against these diseases has also been broadly studied over the last decade. One of the most studied compounds are fat-soluble vitamins, such as vitamin D, which modulate inflammatory responses via the regulation of proinflammatory gene expression, transcription factors and the activation of signaling cascades that mediate inflammatory responses [7]. Other antioxidant compounds like β-carotene and flavonoids play a key role in IBDs by limiting the production of free radicals in cells, inhibiting lipid oxidation or limiting the synthesis of proinflammatory cytokines [8]. Non-starch polysaccharides, classified as dietary fiber, have also been studied extensively as therapeutics against inflammation and other immune-related problems. For instance, Lean et al. treated mice with fucoidans, a compound found in edible brown macroalgae, and observed a reduction in colitis symptoms, diarrhea and the relative weight of the colon, reducing inflammation and edema [9]. Additionally, research evidence suggests that other bioactive food compounds including peptides [10], polyphenols [11] and essential oils [12] exhibited anti-inflammatory and antioxidant effects in animal models of colitis. However, they rarely exist in sufficient amounts in foods to exert the necessary effect [13]. Therefore, the fortification of food products with these nutraceuticals may constitute a simple way of delivering them in sufficient amounts in diets. In fact, there has been an increasing research interest in the development of functional foods/beverages enriched with different nutraceuticals throughout recent years [1]. However, many nutraceuticals often exhibit several limitations, including low water solubility and stability, interactions with the food matrix, low bioavailability, poor absorption and even chemical transformation during digestion, which strongly limit their direct incorporation into food products [1]. To this end, we have previously developed a lupin protein concentrate (LPC) from *Lupinus albus* seeds that contains high amounts of a bioactive polypeptide oligomer named deflamin (patent WO/2018/060528), one of the few existent protein matrix metalloproteases (MMPs) inhibitors (MMPIs) derived from staple foods that effectively reduces gelatinolytic activity in vivo [14]. MMPs comprise a family of zinc-dependent endopeptidases with a well-recognized key role in intestinal inflammation [15], particularly gelatinase MMP-9, which has been shown to be up-regulated in human colitis and other IBDs and is positively correlated with disease severity [15]. It has been shown that MMP-9 inhibition can reduce IBD development [15], meaning that deflamin may hold great potential to be used in functional diets. When administered orally, isolated deflamin reduced colitis lesions, whilst inhibiting MMP-9 activity in mice [14]. In our previous reports, the deflamin-enriched LPC was added to low gluten and gluten-free cookies (10 g protein/100 g cookie dough) [16]. These studies showed that some of the flours used strongly interfered with deflamin activity. Although the LPC could be a very promising delivery system for functional foods against gastrointestinal diseases, it was never tested in vivo. Being a lupin concentrate per se, it should be expected that the other components of the LPC, such as polyphenols and other proteins, might assist the bioactivity of these cookies as well, particularly by reducing oxidative stress [17]. Evidence suggests that IBDs are associated with an imbalance between ROS and antioxidant activity, which generates oxidative stress as the result of either ROS overproduction or a decrease in antioxidant activity [18,19], meaning that the main key players in ROS scavenging are often identified as important clinical targets in IBD management [17,18,19]. Recent reports demonstrate that lupin is a promising source of antioxidant phenolics for functional food production, as well as an inductor of a healthier gut microbiome [20]. These observations highlight the importance to further pursue the potential of this LPC as a functional ingredient, and to test its potential in vivo, both as an MMP-9 inhibitor and as an oxidative stress reducer in IBDs. The present study aimed to evaluate the nutrient and antioxidant value of the LPC, to test its efficacy as a food additive for wheat cookies in reducing colitis in vivo, using two different models of IBD, and in inhibiting MMP-9 and other inflammatory pathways, including oxidative stress.

## 2. Materials and Methods

### 2.1. Biological Materials

The biological material used for the present study was the quiescent seeds of sweet lupin (*Lupinus albus* L.), cv Amiga (Jouffray-Drillaud, Cissé, France). The human colon adenocarcinoma cell line, HT29 (ECACC 85061109), obtained from a 44-year-old Caucasian female, was also used throughout this work.

### 2.2. Preparation of the Lupin Protein Concentrate (LPC)

Approximately 100 g ± 0.1 g of dry lupin seed protein was extracted using milli-Q water (1:10, *w*/*v*). The homogenate was filtered through miracloth (Ref: 475855, Merck Millipore, Burlington, MA, USA). The filtered sample was subsequently boiled for 10 min and filtered again through miracloth. The protein sample was finally freeze-dried (Edwards Micro Modulyo, Crawley, UK) and stored at −80 °C.

### 2.3. Chemical Characterization of LPC

The approximate chemical composition of the lupin extract was calculated based on powdered samples. Total protein determination was evaluated by Dumas Nitrogen Analyser NDA 702 (Velp Scientifica, Usmate Velate, Italy) and the conversion factor used was 5.7. Crude fat was measured using ether extraction according to AOAC 2003.05 and Mota et al. [16]. Ash content, representing the inorganic fraction of the LPC, was measured by incineration at 550 °C in a muffle according to AACC 08-01.01. Moisture content was determined gravimetrically, according to Mota et al. [16]. Total carbohydrates were calculated as (100 − [protein + fat + ash + water content]).

### 2.4. Antioxidant Activity of LPC

The total phenolic content (TPC) of the LPC was assessed following the method described by Batista and colleagues [21], with minor alterations. LPC phenolic compounds were extracted in methanol in a ratio of 1:5 (*w*/*v*). The homogenate was stirred for 1 h in the dark at 4 °C, followed by centrifugation (12,000× *g*, 4 °C, 30 min). The LPC extract or different concentrations of gallic acid (10 μL) were added to Folin–Ciocalteu reagent (Ref: F9252, Sigma-Aldrich, St. Louis, MO, USA) (100 μL at 0.1 M), thoroughly mixed with 80 μL of 7% (*w*/*v*) sodium carbonate, incubated in the dark for 15 min at room temperature and the absorbance measured at 630 nm. The scavenging effect of LPC was determined using the DPPH (2,2-diphenyl-1-picryl-hydrazyl-hydrate) methodology, and the LPC extract reducing power was evaluated by applying the ferric ion reducing antioxidant power (FRAP) method, both described by Batista et al. [21]. Tests were performed in triplicate for each antioxidant activity assay to guarantee reproducibility of the results.

### 2.5. In Vivo Mouse Model of TNBS-Induced Colitis

Six-week-old male CD-1 mice were purchased from Charles River. 2,4,6-Trinitrobenzene sulfonic acid (TNBS, Ref: P2297, Sigma-Aldrich; 2.5% *w*/*v*) in 50% (*v*/*v*) ethanol was instilled as an intracolonic single dose, as previously described [14]. On the induction day (day 0), the mice were anesthetized with ketamine 100 mg/kg + medetomidine 10 mg/kg. Then, 100 µL of TNBS solution was administered through a catheter, carefully inserted 4.5 cm into the colon. The mice were kept for 20 to 30 min in a Tredelenburg position to avoid reflux. The experimental groups included control animals (*n* = 3) without colitis induction, vehicle (Col; *n* = 5), orally administered LPC (0.1 g/kg; p.o. 1×day, for 4 days; LPC-1; *n* = 5), orally administered LPC (1 g/kg; p.o. 1×/day, for 4 days; LPC-2; *n* = 5) and orally administered LPC (10 g/kg; p.o. 1×/day, for 4 days; LPC-3; *n* = 5). Oral administration was performed by oral gavage, daily, starting at 4 h after colitis induction. At day 4, mice were sacrificed by an administration of 0.25 mg/kg BW sodium pentobarbital. At necropsy, blood was collected by cardiac puncture into centrifuge tubes, and serum was stored at −20 °C for further use. Colons were removed, measured in length and observed for diarrhea severity classification. An extraction of colon proteins was performed according to Castaneda et al. [22], with some modification. Briefly, snap-frozen samples of colon were homogenized with liquid nitrogen and extracted in ice-cold extraction buffer (2.5 mL/g tissue) containing 100 mM Tris-HCl, 100 mM NaCl, 100 mM CaCl_2_ and 0.05% Briji 35 (pH 7.6). After 10 min on ice, colon protein extracts were centrifuged for 10 min at 13,000× *g* at 4 °C, the supernatants were collected and stored at −80°C until assayed.

#### 2.5.1. Disease Activity Index (DAI)

To evaluate colitis severity, the disease activity index (DAI) was assessed at the end of the experiment (day 4). DAI was calculated based on clinical sign scoring, including weight loss and stool character, as described by Maheshwari, Balaraman, Sailor and Sen [23].

#### 2.5.2. Total Gelatinolytic Activity

To evaluate MMP-9 activity in colon samples, MMP-9 inhibition was tested using the DQ-gelatin assay (Ref: E12055, Thermo-Fisher Scientific, Waltham, MA, USA), as previously described by Lima et al. [24]. All assays were performed in triplicate.

### 2.6. Cookie Preparation

#### 2.6.1. Savory Cookies

Savory cookies were prepared using 61.5% wheat flour (T55), 1% salt, 1.5% baking powder, 7.5% sunflower oil and 28.5% water, according to a previously optimized model formulation [16,21]. Batches (100 g) were prepared and the ingredients mixed for 1 min on position 4 in a food processor (Bimby, Vorwerk, Wuppertal, Germany). The cookies were molded using 2, 4 and 6 positions on a pasta roller system (three times each position). A square mold was used to cut the laminated dough. The savory cookies were baked in a forced-air convection oven (Unox, Cadoneghe, Italy) at 180 °C for 10 min and were dried in the chamber at 60 °C for 30 min. Finally, the cookies were cooled to room temperature for 30 min, stored in hermetic containers and protected from light.

#### 2.6.2. Sweet Cookies

The sweet cookies were prepared using 57% wheat flour, 15% white sugar, 1% baking powder, 18% margarine and 9% water [25]. Batches (100 g) were prepared and the ingredients mixed for 15 s at a speed of 4 in a food processor (Bimby, Vorwerk, Wuppertal, Germany). The sweet cookies were molded in a circular mold and baked at 110 °C for 40 min in a forced-air convection oven (Unox, Cadoneghe, Italy). After cooling for 30 min at room temperature, the cookies were stored in hermetic containers and protected from the light.

Ingredients were added in identical quantities in all cookie samples, except for the flour, which was substituted by 10% of lupin extract in the LPC cookies.

### 2.7. In Vitro Colon Cancer Cell Assays

#### 2.7.1. Testing LPC Bioactivity in Cookies

The total soluble protein content of each cookie sample (with and without LPC) was extracted with 100 mM Tris-HCl buffer pH 7.5, in a ratio of 1:4 (*w*/*v*). This solution was stirred overnight at 4 °C. Samples were centrifuged in a Beckman J2-21M/E centrifuge at 12,000× *g* for 30 min at 4 °C, and the supernatant was collected and stored at −20 °C.

#### 2.7.2. Wound Healing Assay

HT29 cells were maintained in RPMI medium (Ref: R5886, Sigma-Aldrich) supplemented with 10% fetal bovine serum (Ref: F9665, Sigma-Aldrich), 2 × 10^4^ UI/mL penicillin and 20 mg/mL streptomycin at 37 °C, in a humidified atmosphere of 5% (*v*/*v*) CO_2_. Cell migration was assessed by wound healing assay. HT29 cells (5 × 10^5^ cells/well) were seeded in 24-well plates and allowed to reach 80% confluence. Simulated wounds were performed as described by Lima et al. [24]. Cells were washed twice with PBS to remove debris. Each well was filled with fresh media containing the cookie protein extracts (100 μg/mL). After 48 h, the invaded area was measured for each treatment and compared to the corresponding area at 0 h to determine the area covered de novo by the migrating cells. MMP-9 activity was determined as previously described (Section 2.5.2). In each experiment, both positive (without LPC) and negative (without enzyme) controls were included for all samples to correct possible proteolytic activities present in the LPC extract. All data were corrected by subtracting their corresponding negative controls.

### 2.8. In Vivo Assays Using an AA-Induced Colitis Model

#### 2.8.1. Colitis Induction and Experimental Groups

Male Wistar rats were housed in the vivarium of Universidade Luterana do Brasil (ULBRA, Porto Alegre, Brazil) under a 12 h light/dark cycle and kept at 22 °C ± 2 °C with 55–60% humidity. Water and food were provided ad libitum. Animals weighing an average of 350 g each were divided in five groups: control (Co), control + LPC cookie (Co + LPCc), colitis (Col), colitis + control cookie (Col + Cc) and colitis + LPC cookie (Col + LPCc). The colitis induction model used was adapted from Hartmann et al. [26]. The animals were anesthetized intraperitoneally with a mixture of 50 mg/kg xylazine and 100 mg/kg ketamine hydrochloride. Colitis was induced by intracolonic administration of 4% acetic acid (AA), and the groups received 4 g of LPC cookie or control cookie orally, by gavage, 3 h after colitis induction, daily, for 3 days. Finally, the animals were euthanized by exsanguination under anesthesia and, after necropsy, a portion of the colon (8 cm) was removed. The colons were homogenized with 9 mL of phosphate buffer (KCL 140 mM, phosphate 20 mM, pH 7.4) per gram of tissue. The protein concentration was determined according to the Bradford method [27].

#### 2.8.2. Anal Sphincter Pressure Measurement

The anal sphincter pressure measurement was performed prior to euthanasia. For this procedure, the animals were anesthetized, and manometry was performed using a balloon catheter of water. The result is expressed in cm H_2_O. Three pressure measurements were made for each animal [26].

#### 2.8.3. Lipoperoxidation

Lipid peroxidation was measured by thiobarbituric acid reactive substances (TBARS), measuring the amount of substances that react with thiobarbituric acid (TBA). All tissue samples were mixed with 10% trichloroacetic acid (TCA) and 0.67% TBA, heated at 100 °C for 15 min and cooled in ice. The samples were centrifuged at 1500× *g* for 10 min and 4 °C. The absorbance was determined using a 96-well plate reader at 535 nm [26].

#### 2.8.4. Activity of Antioxidant Enzymes

The activity of superoxide dismutase (SOD), defined by its ability to inhibit the reaction of superoxide radicals with adrenaline, was monitored spectrophotometrically at 560 nm. Results were expressed as USOD/mg protein [28]. The activity of glutathione peroxidase (GPx) was assessed by the NADPH oxidation rate in the presence of reduced glutathione and glutathione reductase. Sodium azide was added to inhibit catalase activity. GPx activity was measured spectrophotometrically at 340 nm and expressed as nmol/min/mg protein [28].

#### 2.8.5. Alkaline Comet Assay

The comet assay was performed as described by Vercelino et al. [29]. The slides were neutralized, stained and analyzed according to Nadin et al. [30]. Images of 100 randomly selected cells were observed from each animal (*n* = 5 per group) and scored according to tail size in five classes, ranging from undamaged (100 × 0) to maximally damaged (100 × 4), resulting in a single DNA damage score for each animal and, consequently, for each group. The damage frequency (%) was calculated based on the number of cells with tail versus those with no tail.

#### 2.8.6. Histopathological Analysis

After the death of the animals, their colons were removed and fixed in 10% buffered formalin for 24 h. Paraffin blocks were sliced with a rotary microtome to create 3 mm thick sections. The tissues were stained with hematoxylin and eosin. The slides were photographed using a NIKON Labophot binocular microscope at a magnification of 200×. The histological analysis was based on changes in the crypts and the presence of inflammation in the colon [26]. The expression of COX-2 and TNF-α proteins in intestinal tissues was determined by immunohistochemical analysis, as described by Hartmann et al. [26]. The slides were analyzed using a microscope equipped with a digital camera using Image-Plus software (Media Cybernetics, Bethesda, MD, USA). The quantification of COX-2 and TNF-α expression was performed via digital analysis with ImageJ and involved counting the positive pixels stained by immunohistochemical analysis with the IHC profiler plugin.

### 2.9. Statistical Analysis

All experiments were performed with at least six replicates in three independent times, and the data are expressed as the mean ± standard deviation (SD) in the case of normal distributions, and as the mean and maximum and minimum levels when data did not follow a normal distribution. Data analysis was performed using the SigmaPlot software (version 12.5). In order to carry out the inferential analysis, and considering the fulfillment of the necessary criteria for the performance of parametric tests, the Kolmogorov Smirnov normality test was performed. In the cases where the samples did not follow a normal distribution, non-parametric tests were used.

For in vitro assays analysis, and given that the sample presented a normal distribution, a one-way analysis of variance (ANOVA) was performed using Tukey’s test to compare the differences between groups, with *p* values less than 0.05 considered statistically significant. 

For in vivo experiment analysis, a comparison between groups was performed by one-way analysis of variance, followed by the Student Newman–Keuls procedure. *p* < 0.05 was considered statistically significant.

## 3. Results and Discussion

### 3.1. Lupin Protein Concentrate (LPC) Holds Nutritional Value and Antioxidant Potential

In our previous works, a lupin protein concentrate (LPC) was developed which contained deflamin, a potent matrix metalloproteinase (MMP)-9 inhibitor that could efficiently reduce colon cancer cells’ migration and MMP-9 activity in vivo and in vitro [14,24]. Although deflamin has been demonstrated to be a potential nutraceutical for IBDs [11], a diet rich in lupin seeds does not seem to be enough for it to exert sufficient activity in IBDs. In addition, some additional components of lupin such as fibers, phytate and protease inhibitors (among others) can hamper digestion and are unadvised for patients suffering from IBDs [5,6]. Under this context, an LPC could achieve significant importance as a bioactive food additive in functional foods, since it contains a higher concentration of deflamin, without most of its anti-nutritional factors [31]. Besides exerting a dose-dependent inhibition of MMP-9 in vitro [14], the LPC presents the extra advantage of being a prepared food, rather than a purified or isolated compound (nutraceutical of pharmacological), which allows it to be characterized as GRAS while permitting an easier application in functional diets [16]. As a lupin concentrate, it has the additional advantage of containing other beneficial health activities due to the presence of specific polyphenols and carbohydrates from lupin seeds which have been reported to reduce oxidative stress and improve the gut microbiome, both rather important factors in reducing or preventing IBDs [20]. Nonetheless, although LPC was tested against MMP activity [16], its nutritional profile and antioxidant activities remained to be studied. Under this context, we initially set out to evaluate the nutritional profile of the LPC, including the phenolic content, and also test its antioxidant potential. The results obtained are shown in Table 1. It is noteworthy to highlight that we used sweet white lupin (*Lupinus albus*) cv. Amiga, whose seeds present little or no alkaloids, thus abolishing the toxicity when compared to bitter lupin seeds.

Being a water-soluble protein concentrate, we expected to find that LPC is a low-fat food product with a high level of protein and water-soluble carbohydrates, as demonstrated in Table 1. Previous reports have shown that water soluble protein extracts from lupin, particularly after boiling, are free from phytate and lectins, which allows it to be more digestible [31].

The presence of a high amount of protein as well as water-soluble carbohydrates suggests a good application of the LPC as a nutritional food additive. Indeed, several authors have pointed lupin as a new and valuable source of protein to nutritionally supplement traditional foods [32], both in animal and human nutrition, and even as an alternative to soybean [32].

The presence of phenolic acids and flavonoids in lupin seeds has been reported in many studies, although when compared to other members of the lupin genus, *L. albus* has been demonstrated to have a lower amount of phenolic compounds (212.12 to 491.51 mg/100 g d.m. as gallic acid equivalents) [32]. In our measurements, the total amount of phenolic compounds was, expectedly, even lower, at around 35 g/100g d.m., since we can only obtain the water-soluble polyphenols in the LPC. Nonetheless, data show that the LPC still holds antioxidant capacity, as measured by the DPPH and the FRAP methods. The main therapeutic effect associated with the antioxidant activity of phenolics mainly protects an organism against the damaging effects of the active oxygen species and free radicals that initiate oxidative processes [32]; hence, these results corroborate that the potential of the LPC against IBDs as an antioxidant agent (besides the MMP-9 inhibition) should also be pursued.

### 3.2. LPC Reduces the Clinical Characteristics of Ulcerative Colitis, in a Dose-Dependent Manner

**Figure 1:**
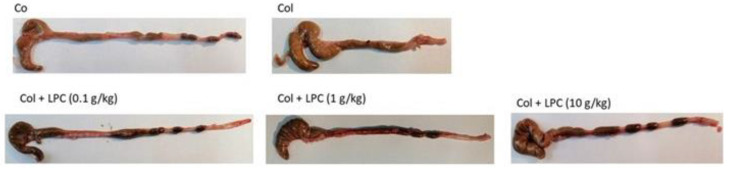
Representative images of the effect of a 4-day lupin concentrate feed in TNBS-induced colitis in mice. LPC was administered orally in three different concentrations (0.1, 1 and 10 g/kg). Co: control group (healthy); Col: colitis group; Col + LPC: colitis group fed with LPC (at each concentration).

Since lupin is already widely used in food production, particularly as a technologically desirable additive in bakery products as well as in dietary and functional food products [32], it seems plausible that our LPC could be considered a promising new addition to the lupin food additives already in the market. However, although previous works have shown that LPC contained anti-MMP-9 activity in vitro, its activity against IBDs remained to be tested in vivo. As previously reported, the effectiveness of nutraceuticals in providing therapeutic or physiologic benefits depends heavily on preserving their bioavailability [1], which may be compromised by several factors such as gastric residence time, low permeability and/or solubility within the gastrointestinal (GI) tract, and instability during food processing/storage or in the GI tract [1]. Although orally-fed deflamin was shown to be resistant to digestion and effectively reduced colitis in TNBS-induced colitis in mice [14], it is possible that the presence of other components in the protein concentrate could interfere with its bioactivity and resistance to digestion. In fact, it has been reported that most nutraceutical activities can be altered by their delivery system, often via interactions with other bioactive molecules [1]. Therefore, before using the LPC as a functional food additive, its dose-response was required to be tested to establish a proper daily dose for functional foods. Therefore, we tested the LPC in vivo in mice using three different concentrations, ranging from 0.1 to 10 g/kg, with the goal of (a) testing its efficacy in vivo and (b) determining the best LPC dose to be used in functional foods and diets. Figure 1 shows the representative images of each colon group, whilst Table 2 shows the disease activity index (DAI) scores, colon lengths observed as well as the MMP-9 activity in colons (Table 2) in mice with TNBS-induced colitis, both in control groups and in groups which were fed daily with three different LPC concentrations.

The results show that the DAI scores were significantly enhanced in the colitis group when compared to the control (healthy) group, but were significantly and consistently lower in all three LPC concentrations (Table 2), in a dose dependent manner, being reduced by more than 50% in treatments with the 10 g/kg LPC administration when compared with the untreated colitis group. A similar impact of LPC was observed in colon lengths. As expected, the colon length in the TNBS group was significantly reduced in comparison with the healthy group (Figure 1, Table 2). With the LPC oral administrations, colon length was significantly higher, also in a dose dependent manner, being significantly higher in the groups treated with 1 and 10 g/kg LPC when compared to the colitis group (10.4 ± 0.2 and 7.23 ± 0.7 cm in the highest LPC concentration and controls, respectively) and overall more significantly similar to healthy individuals (11.2 ± 0.4 cm) (*p* < 0.001). Concerning the observed colon MMP-9 activity (Table 2), results also show a similar trend. Predictably, the untreated colitis group showed an elevated MMP-9 activity when compared to the control, which was consistently reduced with increasing doses of the LPC (*p* < 0.001). Whilst the lower dose of 0.1 g/kg of LPC reduced 20% of MMP-9 activity, the higher doses induced a reduction of 29% and 37% for 1 and 10 g/kg LPC, respectively. The results, therefore, suggest that the LPC contains enough activity to significantly reduce colitis and colitis-induced MMP-9 activity in vivo in dosages higher than 1 g/kg LPC per day to obtain the desired effects in reducing colitis lesions. In similar studies performed with persimmon, pennyroyal and spearmint phenolic extracts in TNBS-induced colitis in mice, an attenuation of histological features, inflammatory markers and reduction in lesion extent in the colon were also observed, corroborating the efficacy of these bioactive compounds [33,34,35]. It is, nonetheless, to the best of our knowledge, the first time that a protein concentrate has been shown to be effective against colitis.

### 3.3. LPC Bioactivity, When Used as a Food Additive, Is Influenced by the Presence of Sugar

In our previous work, we tested the deflamin activity stability of LPC in different types of flours. LPC (from a technological point of view, a concentration of 10 g protein/100 g cookie was selected as the best quantity) was added to gluten-containing and gluten-free flours (rice and buckwheat) to produce savory cookies. The results obtained showed that most low-gluten and gluten-free flours were not the best vehicle to deliver bioactive deflamin [16] since they reduced its activity. Only buckwheat flour showed a better stability of MMP-9 inhibitory activity [13]. Knowing that wheat is a preferred flavor in food markets, and considering that LPC has not been tested in this type of flour, in the present work we opted to incorporate LPC in wheat cookies in sufficient amounts to reach the desired daily dose: above 1 g LPC/kg of body weight. Our main goals were to develop a food product that was well accepted by consumers, cost-effective and with a structure allowing the incorporation of higher levels of LPC. Wheat flour, having better viscoelastic properties, could also facilitate the incorporation of greater amounts of LPC without impairing its technological features, in addition to being more cost-effective than gluten-free flours.

**Figure 2:**
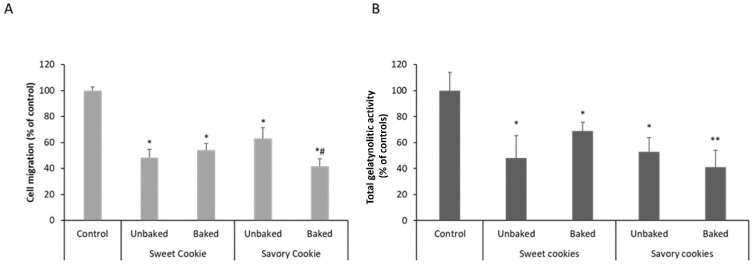
LPC bioactivity in wheat cookies against (**A**) colon cancer cell migration and (**B**) MMP-9 activity. Sweet and savory cookies were tested, both baked and unbaked dough. Data are expressed as the means ± SD. (* *p* < 0.05, ** *p* < 0.001 when compared to control; ^#^ *p* < 0.05 when compared to unbaked savory cookie).

Savory and sweet cookies were baked with the addition of LPC to the wheat flour, and their activities were tested both against MMP-9 and the migration rates of HT29 colon cancer cells. Both cooked and uncooked doughs were tested and the results are shown in Figure 2.

Previous results [16] demonstrated that the nature of the flour could interfere with the activity of deflamin, but that the baking process itself would not. This may derive from the observation that deflamin is resistant to boiling (patent WO/2018/060528). According to Mota et al. [16], the amount and type of sugars present in different flours apparently interfere with the activity of deflamin in LPC. In the present study, the results show that the bioactivity of LPC was maintained with the wheat flour, both in inhibiting MMP-9 (Figure 2B) and in reducing colon cancer migration (Figure 2A). However, interestingly, results differed significantly (*p* < 0.05) between savory and sweet biscuits after baking. Although both activities were maintained in unbaked dough, after baking the sweet cookies have significantly less inhibitory activity (Figure 2A,B), both in MMP-9 inhibition and in the ability to reduce cancer cell migration.

These results suggest that wheat flour per se does not interfere with LPC bioactivity, as previously demonstrated for gluten-free flours [16] but baking in the presence of sugar does. Previous experiments conducted on different types of flours suggested that different amounts of starches and sugary molecules could interfere with deflamin activity when LPC was used as a food additive. This hypothesis is consistent with our results on deflamin MS sequencing [14], which showed, in purified deflamin preparations, the presence of β-conglutin fragments, which are known for their lectin-type sugar binding activities. Nonetheless, by comparing unbaked with baked doughs we can see that it was not the presence of sugar per se that reduced the LPC activity, but the baking process, which allows us to infer that perhaps a Maillard reaction, due to the high amount of proteins in LPC, may be responsible for inhibiting deflamin activities. This is also substantiated by the development of a golden-brown color observed in LPC-containing cookies, particularly in sweet cookies, which was also previously reported [16], suggesting the involvement of Maillard reactions. Overall, these results substantiate the importance of the biochemical composition of the food matrix when incorporating nutraceuticals in foods [1] and should be taken into consideration in future studies using LPC as a food additive. On this matter, Szwajgier et al. [36] observed that enriched bread with plant polyphenols can play a role in HT29 cells by the inhibition of the growth and viability of tumor cells. A similar study performed by Gawlik-Dziki and collaborators [37] showed that bread enriched with broccoli sprouts inhibited the proliferation of stomach cancer cells. Nonetheless, important factors such as digestion and bioavailability are also of vital importance for the efficacy of these bioactives and must be also evaluated using in vivo models.

### 3.4. LPC-Enriched Cookies Are Effective as Functional Foods against AA-Induced Colitis In Vivo

Once the LPC-containing cookies were selected, we proceeded to test their anti-inflammatory activity in vivo. Since the concentration of 10 g protein/100 g cookie dough was preferred for yielding the best technological biophysical properties such as texture, LPC cookies had to be fed to animals in sufficient amounts so that at least a dose of 1 g LPC/kg of body weight per day would be assured. This is roughly the equivalent to 4 g of cookies per day, a higher dose than mice could take daily. Therefore, we used a rat colitis model instead, so that the dose per day could be enough to significantly reduce colitis. Since we aimed at testing not only the anti-inflammatory activity but also the antioxidant activity of LPS in vivo, we opted for an acetic acid (AA)-induced colitis. In this way, we could provide information on the effect of deflamin and of LPC on a model of IBD that is very similar to human acute IBD (ulcerative colitis) in terms of pathogenesis, histopathological features and inflammatory mediator profile, as opposed to the acute colitis of the TNBS model [38,39]. AA-induced colitis was found to cause non-transmural inflammation characterized by increased neutrophil infiltration into the intestinal tissue, vascular dilation, necrosis of mucosal and submucosal layers, edema and destruction of crypts [38,39]. Additionally, AA-induced colitis induces higher oxidative stress [26,38], allowing us to better observe any type of antioxidant potential as well. This was particularly important due to the results presented above on the antioxidant potential of LPC (Table 1), and because several studies have revealed that oxidative stress plays a critical role in the initiation and progression of IBDs [19,26,38,39].

**Figure 3:**
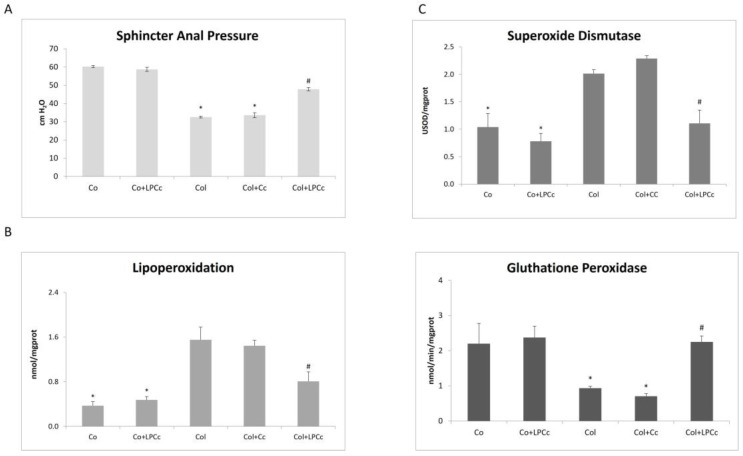
Effect of administration of LPC cookies on rat with acetic acid-induced colitis. Anal sphincter pressure (**A**) and lipid peroxidation (**B**) were analyzed in both controls and diseased rats, as well as the activity of ROS-related enzymes superoxide dismutase (**C**) and glutathione peroxidase (**D**). Values are expressed as the mean ± standard error. Co: control; Co+LPCc: control + LPC cookie; Col: colitis; Col + Cc: colitis + control cookie; Col + LPCc: colitis + LPC cookie. * represents *p* < 0.001 when compared to the control and ^#^ *p* < 0.001 when compared to colitis.

For the reasons outlined above, we tested the effect that a daily dose of 4 g LPC cookies/kg of body weight had on the lesions and oxidative stress levels in rats with AA-induced colitis. Figure 3 shows the effect that the ingestion of LPC-containing cookies had on sphincter pressure (A) and lipid peroxidation (B), as well as on superoxide dismutase (C) and glutathione peroxidase activities (D).

As a way to evaluate the extent of colitis and diarrhea, we measured the anal sphincter pressure using anorectal manometry (Figure 3A). As expected, the colitis group showed a significant decrease when compared to the other groups, due to the muscle relaxation caused by severe diarrhea. However, the animals fed with LPC-cookies showed a significant increase in the sphincter pressure values (*p* < 0.001). Noticeably, rats with colitis fed with the control cookies (with no LPC) had similar values when compared to the colitis group.

We also aimed to evaluate the impact that LPC could exert on the level of oxidative damage induced by colitis, as determined by the level of lipid peroxidation (LPO) in the colon tissues of the different animal groups (Figure 3B). As expected, there was a very significant increase in LPO levels in the colitis group. As observed for the anal pressure measurements, no differences between the colitis group and the Col+Cc group were observed, suggesting that there was no impact on LPO when the rats were fed with wheat cookies. However, a significant reduction in LPO (*p* < 0.001; Figure 3B) was detected in the LPC-cookies treatment group, which was reduced by 40% when compared to the colitis group. This significant reduction in oxidative lesions, along with the reduction in anal sphincter pressure, suggests that there was a potent protective effect of LPC on colitis.

It has been well established that, in IBD patients, the production of reactive oxygen species (ROS) is increased [23,39], mostly because the infiltration of neutrophils leads to the production of superoxide anion (O_2_) and initiates the production of various ROS that significantly contribute to the progression of tissue necrosis and mucosal dysfunction [17,19,39]. Several enzymes, such as SOD and GPx, prevent the accumulation of O_2_ and hydrogen peroxide (H_2_O_2_) and are, therefore, considered the primary line of defense. We further analyzed the impact of LPC cookies on the activities of SOD and GPx, and results are depicted in Figure 3C,D, respectively. In our study, we observed a significant increase in SOD activity in the colitis group (Figure 3C), most likely to compensate for the damage caused by the action of acetic acid in the animals’ intestines. The enzyme SOD has an essential role in cellular redox balance, promoting dismutation in an attempt to free radicals, protecting tissues against oxidative damage [28]. However, whilst the Col + Cc group presented similar levels (*p* < 0.001) of SOD activity when compared to the colitis groups, the treated group showed a significant decrease in SOD activity, even reaching levels that were significantly similar to the control (healthy) group. This trend was also consistent with the activity of GPx (Figure 3D), which was reduced in the colitis group as well as the Col + Cc treatments (*p* < 0.001), but was restored with the LPC cookie administration compared to the colitis group, once again reaching similar levels to those observed in healthy animals. Glutathione peroxidase (GPx) has a great physiological importance because it catalyzes the decomposition of inorganic peroxide and organic peroxides using glutathione (GSH) as a co-substrate, which is a key component protecting against damage by free radicals in the physiological system [40]. Several studies have shown that GPx is responsible for H_2_O_2_ detoxification when it is present at low concentrations [41], and the reduction of GPx activity in the intestines of animals with colitis is known to be blocked after the administration of antioxidants [41,42]. Similar results were demonstrated by Tahan et al. [43], who administered doses of melatonin in a colitis model in rats and also observed an increase in the enzyme GPx. This corroborates the results found in our study.

Overall, considering the oxidative stress levels, our results showed that the oral administration of LPC-containing cookies ameliorated the symptoms induced by colitis and consistently reduced the damage and enzymatic activities related to oxidative stress, restoring the levels of ROS-related enzymes to healthy levels. These results further substantiate that LPC, besides reducing MMP-9 activity, also affects the oxidative stress induced by colitis. More importantly, its activity was not reduced by the digestion process, which often occurs with nutraceuticals [44]. Previous reports showed that the activity of deflamin isolated from lupin seeds was maintained when it was orally administered in animal models of disease [14] and was able to significantly reduce colitis and gelatinase activity. However, this is the first report that shows the maintenance of deflamin activity in LPC after digestion, as well as of the anti-oxidative activity of LPC itself. It is important to note that deflamin per se does present noticeable antioxidant activity (data not shown). Hence, the LPC combines antioxidant activity with the MMP-9 inhibition by deflamin, elevating the potential of this food additive. 

**Figure 4:**
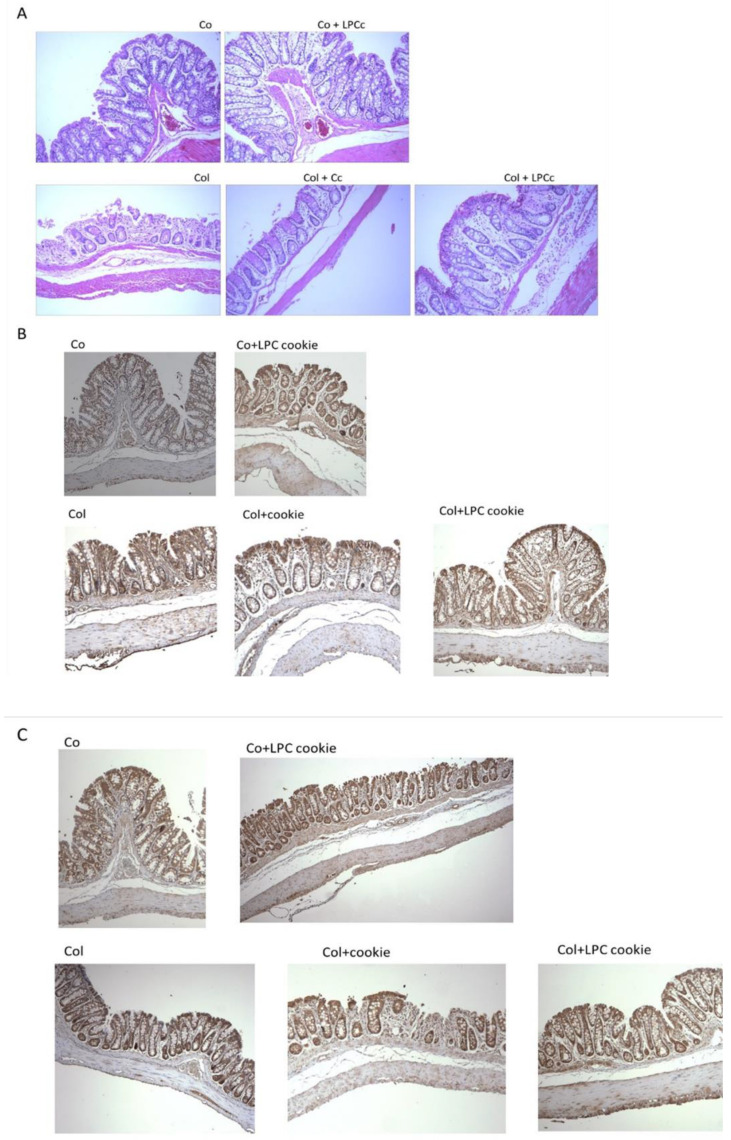
Effect of administration of deflamin cookies on colon tissues. (**A**) Histopathological alterations in colitis in colon tissue. (**B**) Immunohistochemical detection of COX-2. (**C**) Immunohistochemical detection of TNF-α. Co: control; Co + LPCc: control + LPC cookie; Col: colitis; Col + Cc: colitis + control cookie; Col + LPCc: colitis + LPC cookie. All images are at a magnification of 100×.

To better evaluate the protective effects of LPC in AA-induced colitis, we further assessed the impact of LPC cookies on the histological lesions induced by colitis, as well as on the expression of important biomarkers of inflammation, COX-2 and TNF-α. The results are depicted in Figure 4. Table 3 presents the expression of the positive pixels of each colon group.

Figure 4A shows the histopathological alterations in colon tissue with colitis. Representative H&E-stained histological sections showed that the colons from both Co and Co + LPCc groups presented a healthy epithelium with well-organized crypts and no ulceration or tissue erosion. In contrast, the histopathological analysis showed macroscopic and microscopic alterations in the colitis group compared to control groups. The colitis group showed inflammatory lesions, characterized by loss of crypts, surface erosion and ulceration. Notably, in the Col + LPCc group, we observed a significant reduction in overall colon injury, suggesting, once again, a protective LPC effect on histopathological lesions in comparison with the untreated Col group. These results substantiate that LPC can attenuate the tissue changes induced by experimental colitis. This result was expected, as previous studies showed that deflamin could reduce the tissue lesions induced by experimental TNBS-induced colitis, corroborating the maintenance of its activity in LPC cookies. Nonetheless, these results could also be associated, at least in part, with an antioxidative effect. Indeed, studies using antioxidant compounds such as vitamin E and rivastigmine also showed a reduction in the damage caused by agents that are used to induce colitis [45,46]. Therefore, we further analyzed the expression of the biomarkers of inflammation, COX-2 and TNF-α, by IHC. We observed a significant increase (*p* < 0.01) in both biomarkers in colon tissue in the colitis group (Figure 4B,C and Table 3), as opposed to the control (healthy) groups, which did not show positive staining. Treatment with LPC-cookies reduced the expression of both these biomarkers in the treated groups (*p* < 0.01), corroborating the anti-inflammatory effect of LPC (Table 3).

Aiming at further testing the protective effects of LPC-cookies, we evaluated the level of DNA damage induced by colitis in all studied groups using a comet assay. Table 4 shows the results obtained for damage index and damage frequency in peripheral blood.

The data showed a significant increase in baseline DNA damage in the colitis group when compared to the healthy groups, as measured by damage index and damage frequency (Table 4). As in previous assays, cookies supplemented with LPC significantly (*p* < 0.001) decreased the damage induced by colitis, restoring it to values similar to the controls, in both measured parameters. It is noteworthy to observe that in the healthy groups, there was no impact on DNA induced by the LPC cookies, hence substantiating the notion that LPC is a GRAS food product and can be safely used by consumers without any IBD symptoms. This is consistent with previous reports which showed that protein extracts from lupin, as well as isolated deflamin, did not exert any apparent cytotoxicity on HT29 cells [14,24].

### 3.5. Considerations on the LPC Cookies as Functional Foods

Currently, *Lupinus albus* is not only appreciated for its nutritional value but also for its functional properties in bakery and confectionary [47], and for human health. Ingestion of lupin-containing foods has been associated with the prevention of obesity, diabetes, eventually cardiovascular disease and, more recently, digestive tract disease [48,49]. The discovery of deflamin and its potential for IBDs has further enhanced the potential of lupine-based foods. Here, the development of a lupin protein concentrate that has nutritional value, antioxidant activity and also reduces colitis-inflammation whilst surviving digestion, and without exerting any toxic effects, can have a great potential for functional diets against IBDs. The incorporation of LPC in cookies has the added advantage of a longer shelf-life and the ability to serve as vehicle for supplementation with nutraceuticals [13,50]. Evidently, there are several limitations to these types of studies, namely the sample size, the duration of the study, lack of other types of IBD models and the fact that these are animal models and not clinical studies. Nonetheless, the efficacy of the LPC against two different types of colitis and the maintenance of the activity in the colons (*i.e.,* it survived digestion) allow us to speculate on its high potential for future human studies. Currently, there is no effective therapy available capable of treating IBDs and, to date, this is, to the best of our knowledge, the first functional food developed with realistic applications for IBDs. Further studies should, therefore, be pursued in order to bring these types of functional foods onto clinical studies, allowing the assessment of their potential against IBDs to be validated.

## 4. Conclusions

The lupin protein concentrate (LPC) was shown to be effective as a delivery system for deflamin, both as a functional food and as an additive to wheat cookies. Besides its activity towards MMP-9, the LPC further added a high nutritional and antioxidant value to the already potential health benefits of deflamin. Having been shown to be effective against two different types of colitis models, the LPC seems to be a potential functional food to be used in preventive/curative diets against IBDs.

## 5. Patents

DEFLAMIN: Therapeutic protein, CT International Patent Application No. PCT/EP2017/075020. Filed on 30 September 2017.

## Figures and Tables

**Table 1:** Chemical composition and antioxidant capacity of the lupin protein concentrate (LPC). Data are expressed as the mean ± SD.

Component	Amount
Moisture (g/100 g)	10.31 ± 2.53
Total Ash (g/100 g)	7.25 ± 0.27
Crude Fat (g/100 g)	0.31 ± 0.05
Crude Protein (g/100 g)	20.09 ± 0.58
Carbohydrates * (g/100 g)	62.05
Total Polyphenols (mg/100 g)	35.19 ± 2.5
Antioxidant Activity	
FRAP (mg AAE/10 mg LPC)	351.19 ± 2.5
DPPH (mg AAE/ 10 mg LPC)	273.9 ± 2.03

* Carbohydrates were determined by differences in the mean ash, fat, protein and moisture contents.

**Table 2:** Effect of a 4-day lupin concentrate feed in TNBS-induced colitis in mice. Disease activity index (DAI) determined as the average of the score of weight loss and stool consistency; average colon length (cecum until rectum), represented in cm; MMP-9 activity measured by the DQ-gelatin kit. Co: control group (healthy); Col: colitis group; Col + LPC: colitis group fed with LPC (at each concentration). Non-parametric data are expressed as the mean and respective maximum and minimum levels, and parametric data are expressed as medium ± SD. (* *p* < 0.05, ** *p* < 0.001 in relation to colitis and ^#^ *p* < 0.05 in relation to control).

	DAI Score		Colon Length (cm)		Total Gelatinolytic Activity (%)
	Mean ± SD	Min; Max	Mean ± SD	Min; Max	Mean ± SD
Co	0 ± 0	(0; 0)	11.4 ± 0.4	(10.9; 11.8)	0 ± 1.9
Col	3.4 ± 0.4	(2; 4)	7.2 ± 0.77 ^#^	(6.5; 8.2)	100 ± 1.5
Col + LPC (0.1 g/kg)	2.5 ± 0.6 *	(1; 3)	10. 8 ± 2.2	(8.7; 12.8)	83.1 ± 1.7 *
Col + LPC (1 g/kg)	2.2 ± 0.3 *	(1; 3)	9.7 ± 1.3	(8.8; 10.9)	71.6 ± 1.3 **
Col + LPC (10 g/kg)	1.5 ± 0.2 **	(0; 3)	10.4 ± 0.4	(10.1; 10.9)	65.4 ± 1.2 **

**Table 3:** Expression of the positive pixels as the mean ± SD obtained for each colon tissue. Co: control; Co + LPCc: control + LPC cookie; Col: colitis; Col + Cc: colitis + control cookie; Col + LPCc: colitis + LPC cookie. * represents *p* < 0.001 when compared to colitis and ^#^ *p* < 0.05 when compared to control.

	COX-2	TNF-α
Co	0.9 ± 0.3 *	0.7 ± 0.1 *
Co + LPCc	1.94 ± 1.5 *	2.4 ± 0.6 *^,#^
Col	11.7 ± 1.98 ^#^	8.7 ± 1.6 ^#^
Col + Cc	5.1 ± 2.1 *^,#^	2.5 ± 0.6 *^,#^
Col + LPCc	0.9 ± 0.2 *	1.2 ± 0.2 *^,#^

**Table 4:** Comet assay in peripheral blood of the studied groups. Co: control; Co + LPCc: control + LPC cookie; Col: colitis; Col + Cc: colitis + control cookie; Col + LPCc: colitis + LPC cookie. * *p* < 0.001 when compared to control and ^#^ *p* < 0.001 when compared to colitis.

Group	Damage Index	Damage Frequency
Co	17.0 ± 5.7 ^#^	17.0 ± 5.7 ^#^
Co + LPCc	21.4 ± 3.6 ^#^	21.4 ± 3.6 ^#^
Col	68.8 ± 7.9 *	57.5 ± 11.5 *
Col + Cc	40.3 ± 9.2 *^#^	35.3 ± 10.4 *^#^
Col + LPCc	17.4 ± 3.4 ^#^	17.2 ± 3.3 ^#^

## Data Availability

Not applicable.
